# Poor mid-term functional patency and post-operative outcomes in diabetic patients who undergo arteriovenous graft creation

**DOI:** 10.1177/11297298241293493

**Published:** 2024-11-07

**Authors:** Colin M Cleary, Rong Wu, Kwame S Amankwah, Mina L Boutrous

**Affiliations:** 1 Division of Vascular and Endovascular Surgery, University of Connecticut School of Medicine, Farmington, CT, USA; 2 Department of Public Health Sciences, University of Connecticut, Farmington, CT, USA

**Keywords:** Hemodialysis, arteriovenous fistula, arteriovenous graft, diabetes mellitus

## Abstract

**Introduction:**

Diabetes mellitus is a leading cause of renal failure in the US and has been associated with higher mortality when compared to nondiabetic patients. This remains true despite initiation of renal replacement therapy. As such, we were interested in identifying any potential differences in access durability and postoperative outcomes in diabetic patients who receive arteriovenous fistulas versus grafts for hemodialysis.

**Methods:**

Diabetic patients undergoing their first arteriovenous (AV) access creation surgery in the Vascular Quality Initiative from January 2011 to January 2022 were included in our study. After exclusions, the study included two groups: those receiving AV fistulas and those receiving AV grafts for hemodialysis. Demographic characteristics were summarized and compared between these two groups using chi-square analysis or unpaired *t*-test. After propensity score matching was conducted, the effect of procedure type on functional patency, along with secondary outcomes including wound infection were assessed using chi-square analysis.

**Results:**

A total of 20,159 diabetic patients who used their hemodialysis access were included in our study; 16,205 received AV fistulas while 3954 received AV grafts. Patients receiving AV grafts were more likely to be older, female, and have higher pre-operative catheter usage. After propensity score matching, patients who received AV grafts had a shorter time-to-use their conduit (50 vs 166 days, *p* < 0.0001), however, patients who received AV fistulas were more likely to have longer functional patency use for hemodialysis when compared to those who received AV grafts (mean survival time: 3.3 vs 2.9 years, *p* < 0.0001). These results were consistent between diabetics with insulin-dependent or insulin-independent diabetes.

**Conclusion:**

Patients diagnosed with diabetes mellitus had an increased risk for significantly inferior clinical outcomes related to newly created AV grafts, including lower rates of mid-term functional patency and higher rates of worse post-operative outcomes when compared to diabetics who received AV fistulas.

## Introduction

Hemodialysis vascular access dysfunction is a major determinant of morbidity and mortality; hemodialysis patients are twice as likely to be hospitalized than the corresponding age-adjusted general population.^1,2^ The two major conduits that are used regularly for hemodialysis access include arteriovenous fistulas and arteriovenous prosthetic grafts. The process of selecting a conduit based on clinical practice guidelines is based on many factors such as available and adequate autologous vein, patient preference, and medical comorbidities, including obesity, peripheral artery disease, and diabetes mellitus.^3–5^

Diabetes mellitus is one of the leading causes of renal failure in the United States and has been associated with higher mortality in peritoneal and hemodialysis during the first 2 years of treatment compared to nondiabetic patients.^6^ To date, there have been sparse reports regarding hemodialysis access in diabetic patients, including a single-institution retrospective study identifying loss of patency and maturity of antebrachial fistulas.^7^ Another study has utilized the United States Renal Database System from 2007 to 2014 to conclude similar findings, detailing increased loss of functional access of arteriovenous fistulas, but not arteriovenous prosthetic grafts, in diabetic patients.^8^ However, there have been no contemporary studies utilizing a robust large-scale database to compare arteriovenous grafts and fistulas’ long-term utilization, durability, and functional access in the diabetic population. As such, we sought to retrospectively identify diabetic patients in the Vascular Quality Initiative (VQI) database from 2011 to 2022 and compare their peri-, post-operative outcomes and mid-term functional patency for hemodialysis use based on their first access selection.

## Methods

Procedural and long-term follow up data in the Hemodialysis module was received from the Vascular Quality Initiative (VQI) and included data from January 2011 to June 2022. Institutional Review Board approval was not required for this deidentified data, maintained by the Society for Vascular Surgery, however, this study was approved by the National Research Advisory Committee (RAC). Initially, all 68,788 patients were sorted based on their diabetes diagnosis. 26,670 patients did not have diabetes or did not have any designation of diabetic status and were excluded. 197 diabetic patients were also excluded if they were younger than 18 years old, had no age listed in the database, if they did not have any graft type distinction, or if they received endovascular arteriovenous fistulas due to low sample size.

We then excluded patients based on lack of information about functional patency for their first procedure, including not having any information in the following VQI fields that described time-to-use (“1st Use Days,” “Dialysis Start Days”) and/or time-to-abandon (“Abandoned Days,” “Dialysis Fail Days”); this excluded 2412 patients that had none of this information. Partial data was included. Non-first-time surgical procedures for patients were not included in this analysis, however, these patients’ first access procedure and follow up was included. To the best of our ability, this exclusion was based on the abandonment of the existing AV graft or fistula and was not included if there were additional procedures to re-establish existing functional access, like thrombectomy, thrombolysis, or revision. This resulted in a diabetic patient dataset with 20,159 patients, 13,658 (67.8%) of who had insulin-dependent diabetes while the remaining 6501 (32.2%) had insulin-independent diabetes, managed by non-insulin medications and/or lifestyle modifications alone.

**Figure 1.**
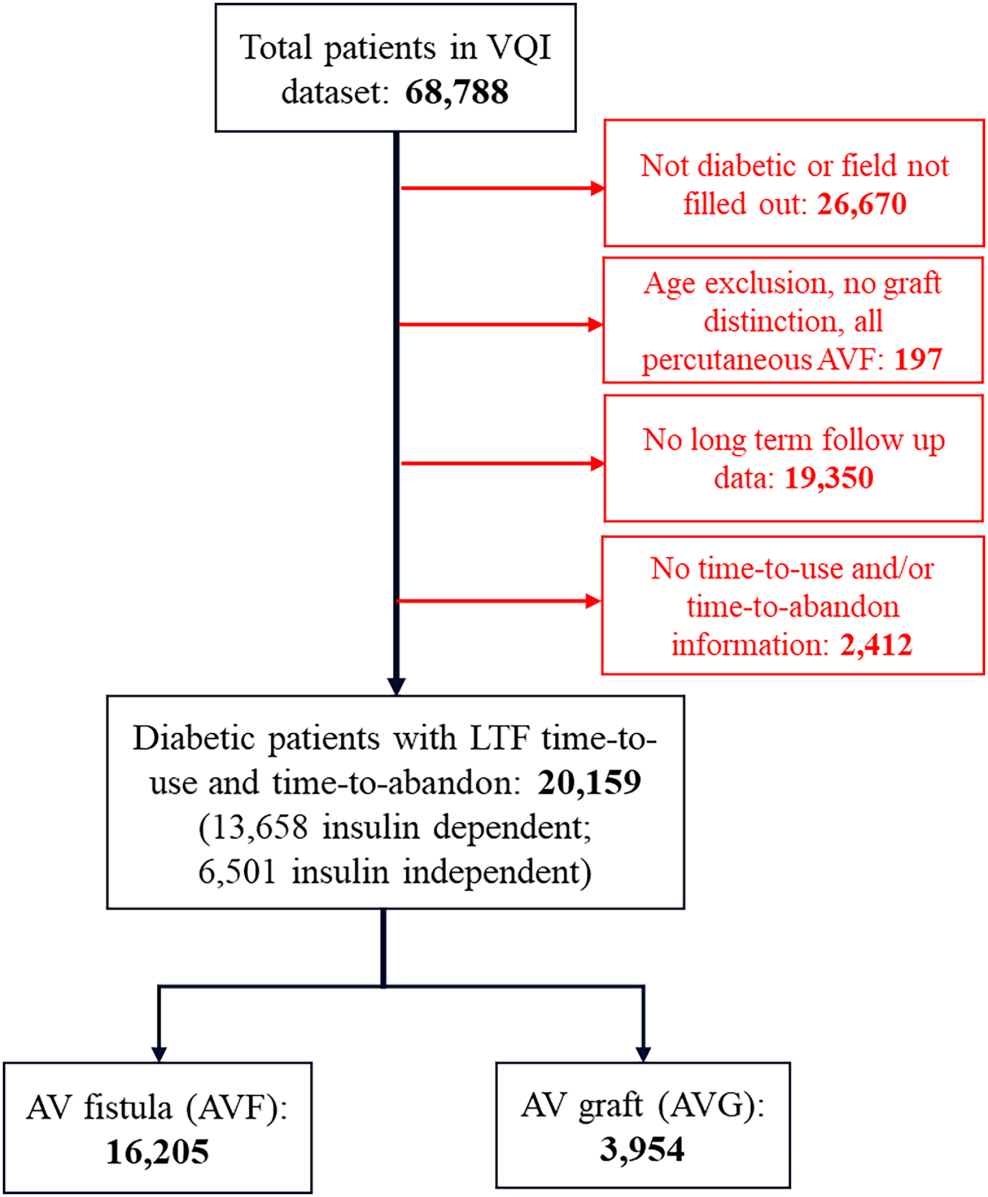
Flow diagram of selection criteria from the VQI hemodialysis module.

In total, 16,205 diabetic patients received an arteriovenous fistula (AVF), while 3954 diabetic patients received an arteriovenous graft (AVG; Figure 1). As described above, our primary outcome measure is “functional patency” for which we utilized the difference of time-to-abandon and time-to-use. As we are interested in functional patency for this study, it is important to note that we have effectively removed all patients that have never used their access with our inclusion criteria. Given that there could be many reasons for never using AV access that are not quantifiable in the VQI dataset, including a change in patient prognosis, continued shared decision making between physician and patient, social factors like adequate access to a hemodialysis facility, or simply the patient does not require hemodialysis yet, among others, we decided to not follow those patients for this study.

### Statistical analysis

All the statistical analyses were conducted in SAS^®^ V9.4 (SAS Institute Inc., Cary, NC), using a two-sided alpha level of significance of 0.05. We first explored the underlying distribution of variables of interest in the two group of patients (e.g. the AV fistula group and AV graft group). The numerical and categorical variables were summarized as means, standard deviations and frequencies, percentages, respectively. For between-group comparisons of numerical and categorical variables, unpaired two-sample *t*-tests and Pearson’s Chi-square tests was conducted, respectively.

In order to reduce the confounding bias in the between-group comparisons of outcome variables, we conducted propensity score analysis using the SAS PSMATCH procedure. The logistic regression model used for propensity score estimation, with receipt of AV grafts as binary outcome variable, included “Gender,” “Race,” “Age,” “BMI,” “Hypertension,” “Smoking history,” “Coronary artery disease,” “COPD,” “Diabetes,” “CHF,” “ASA Class,” and pre-operative catheter use (values 1 and 2 only from the VQI) as predictors. Using the logits of propensity scores created from the above PS model, optimal matching method was used to match patients receiving surgical AV fistulas to patients receiving AV grafts at a 1:1 ratio, with exact matching of gender and caliber of ⩽0.25. The covariate balance in the matched datasets were further assessed. Using the propensity score matched dataset, we conducted Chi-square tests to make between-group comparisons of primary outcomes (e.g. functional patency) and other secondary outcomes (e.g. post-operative course variables). In total, 2,560 diabetic patients per access type were compared in these datasets (5,120 patients total).

## Results

A total of 20,159 diabetic patients underwent arteriovenous (AV) access creation from January 2011 to June 2022; 16,205 (80.4%) received AV fistulas while 3,954 (19.6%) received AV grafts (Figure 1). Demographic information for both groups is listed in Table 1. Patients who received AV grafts were more likely to be older (64.5 vs 61.9 years, *p* < 0.0001), female (56.2% vs 39.1%, *p* < 0.0001), non-Hispanic black (46.9% vs 32.0%, *p* < 0.0001), have a nursing home as a primary residence (6.8% vs 3.4%, *p* < 0.0001), have worse mobility (wheelchair use: 11.3% vs 6.4%, *p* < 0.0001) and use catheter access for dialysis before hemodialysis access creation (58.7% vs 53.1%, *p* < 0.0001). Patients who received AV grafts were also more likely to be a higher ASA class compared to those who received AV fistulas (Class 4: 45.7% vs 38.3%, *p* < 0.0001). Patients who received AV fistulas were more likely to have a higher BMI (31.2 vs 30.0, *p* < 0.0001) and have insulin-dependent diabetes (67.5% vs 65.3%, *p* = 0.012). There were also no differences in smoking history between groups (*p* = 0.24).

**Table 1.** Pre-operative demographics of all diabetic patients who receive AV intervention.

	AV fistula (*N* = 16,205)	AV graft (*N* = 3,954)	*p*-Value
Age, years	62.6 (±12.7)	**64.6 (±13.0)**	**<0.0001**
Female sex	6,647 (41.0)	**2,259 (57.1)**	**<0.0001**
BMI	**31.6 (±8.0)**	30.2 (±8.1)	**<0.0001**
Race			
Hispanic/Latino	**1,678 (10.4)**	337 (8.5)	**<0.0001**
Non-Hispanic Black	4,865 (30.0)	**1,824 (46.1)**	
Non-Hispanic White	**8,139 (50.2)**	1,500 (37.9)	
Other	**1,523 (9.4)**	293 (7.4)	
Co-morbidities			
Insulin-dependent diabetes	**11,045 (68.2)**	2,613 (66.1)	**0.012**
*Smoking history*			
Never	7,981 (49.3)	1,986 (50.3)	0.090
Prior	6,074 (37.5)	1,492 (37.8)	
Current	2,137 (13.2)	470 (11.9)	
Hypertension	**15,648 (96.8)**	3,757 (95.1)	**<0.0001**
Coronary artery disease (CAD)	4,349 (26.8)	1,042 (26.4)	0.56
Congestive heart failure (CHF)	5,549 (34.3)	1,409 (35.7)	0.096
Chronic obstructive pulmonary disease (COPD)	2,435 (15.0)	637 (16.1)	0.091
Catheter access for dialysis	6,818 (44.6)	**2,124 (55.3)**	**<0.0001**
*Living status*			
Home	**15,538 (96.0)**	3,642 (92.3)	**<0.0001**
Nursing home	607 (3.8)	**295 (7.5)**	
Homeless	37 (0.2)	9 (0.2)	
*Pre-operative ambulatory status*			
Ambulatory	**12,781 (92.7)**	2,938 (87.0)	**<0.0001**
Wheelchair	920 (6.7)	**390 (11.5)**	
Bedbound	85 (0.6)	**51 (1.5)**	
Pre-operative aspirin	**1,339 (52.5)**	286 (47.5)	**0.027**
ASA classification			
Class 1	51 (0.3)	12 (0.3)	**<0.0001**
Class 2	406 (2.5)	86 (2.2)	
Class 3	**9,475 (58.6)**	2,067 (52.5)	
Class 4	6,216 (38.5)	**1,765 (44.8)**	
Class 5	13 (0.1)	9 (0.2)	

Shown in table are Mean (±SD) for continuous variables, *n* (%) for categorical variables.

Bold p-values highlight significance (p <0.05)

Given these stark differences in demographics, we utilized propensity score matched patients for further analysis, based on 12 demographic parameters of interest including but not limited to age, BMI, ASA class, and pre-operative catheter use; this resulted in two groups of 2,560 patients who received either conduit. Peri-operative and post-operative measures were compared and are displayed in Table 2. Patients who received AV grafts were more likely to undergo general anesthesia versus regional or local anesthesia (55% vs 28.5%, *p* < 0.0001). Post-operatively, these same patients were more likely to experience a surgical wound infection compared to patients who received AV fistulas (4.5% vs 1.0%, *p* < 0.0001). Patients who received AV grafts also had an increased inpatient post-operative length of stay (1.0 ± 4.6 vs 0.6 ± 3.1 days, *p* < 0.0001) and were more likely to be discharged to any residence other than home including rehabilitation units or nursing homes (6.8% vs 5.5%, *p* = 0.024) compared to patients who received AV fistulas. Although discharge aspirin prescription was similar between groups, rates of anti-coagulant prescription, including warfarin, rivaroxaban, and dabigatran, were increased in patients who received AV grafts (13.8% vs 10.7%, *p* = 0.002).

**Table 2.** Post-operative quality metrics for propensity score-matched diabetic patients receiving AV intervention for hemodialysis.

	AV fistula (*N* = 2,560)	AV graft (*N* = 2,560)	*p*-Value
Operative anesthesia			
Local	**936 (36.7)**	376 (12.7)	**<0.0001**
Regional	**888 (34.8)**	773 (30.3)	
General	727 (28.5)	**1,404 (55.0)**	
Surgical wound infection	16 (1.0)	**76 (4.5)**	**<0.0001**
Total length of stay (mean ± SD)	1.6 (5.8)	**2.7 (24.1)**	**0.016** ^^^
Post-operative length of stay (mean ± SD)	0.6 (3.1)	**1.0 (4.6)**	**0.0005** ^^^
Discharge status			
Home	**2,414 (94.3)**	2,374 (92.7)	**0.24**
Rehabilitation unit	43 (1.7)	**52 (2.0)**	
Nursing home	96 (3.8)	**123 (4.8)**	
Other hospital	4 (0.2)	7 (0.3)	
Homeless	3 (0.1)	4 (0.2)	
Discharge medications			
Aspirin	1,293 (50.6)	1,305 (51.1)	0.71
Anti-coagulants (warfarin, rivaroxaban, dabigatran)	273 (10.7)	**353 (13.8)**	**0.006**
Length of time to follow-up (median days, min–max)	**385 (23–2847)**	373 (10–2369)	**<0.0001** ^^^
Additional procedures after index access creation			
None	**2,455 (95.90)**	2,388 (93.28)	**<0.0001**
1	95 (3.71)	**150 (5.86)**	
2	9 (0.35)	**21 (0.82)**	
3	0 (0.00)	1 (0.04)	
4	1 (0.04)	0 (0.00)	

*n* (%) for categorical variables. .

^ Wilcoxon rank sum test was used for between-group comparison.

Bold p-values highlight significance (p <0.05)

**Figure 2.**
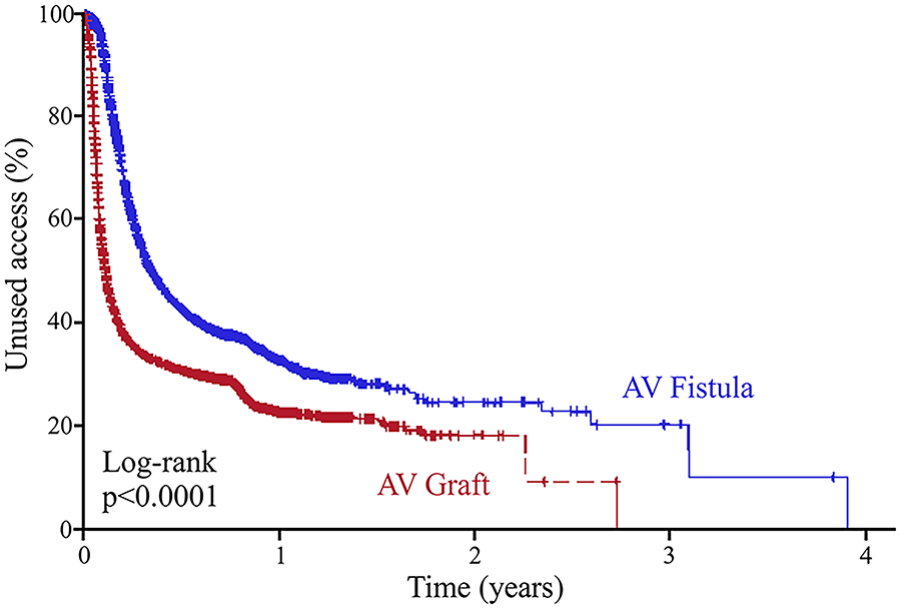
Time-to-use hemodialysis access in propensity score-matched diabetic patients.

**Figure 3.**
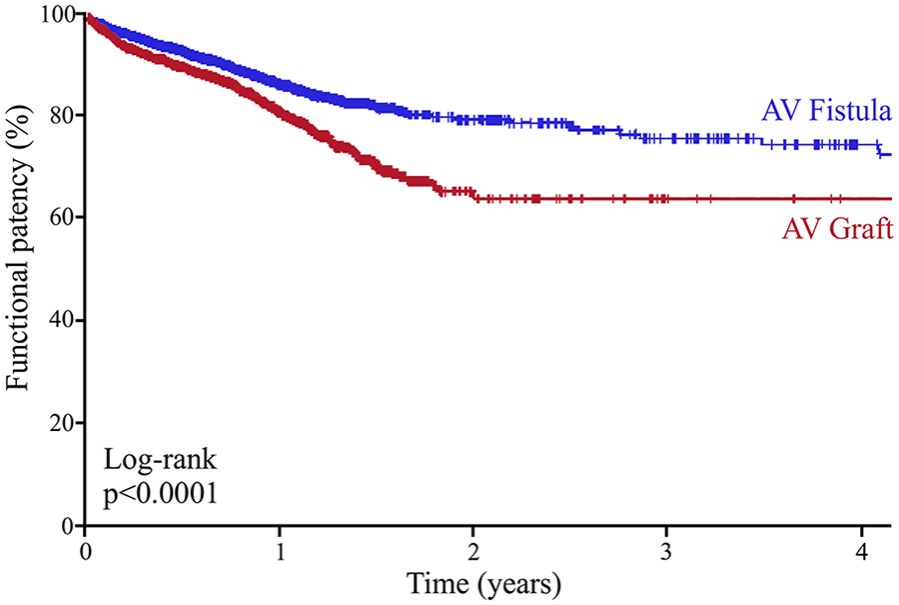
Mid-term functional patency in propensity score-matched diabetic patients receiving AV intervention for hemodialysis.

After discharge, functional patency was assessed by follow up surveillance, including values of time-to-use for hemodialysis as well as time-to-abandonment from hemodialysis. The mean follow-up for both groups was around 1 year, however, patients who received AV fistulas had statistically longer follow up times than those who received AV grafts (385 vs 373 days, *p* < 0.0001, Table 2). Upon follow up, patients who received AV grafts were more likely to use their access earlier for hemodialysis compared to patients who received AV fistulas (median time to use: 50 ± 3 days vs 166 ± 14 days, *p* < 0.001; Figure 2). However, the time of functional patency, as assessed by the difference between time to use and time to abandonment, was higher for patients who received AV fistulas compared to those who received AV grafts (mean years of functional use: 3.28 vs 2.90 years, *p* < 0.001; Figure 3). We also compared the propensity score matched patient population for additional procedures to create subsequent AV access at different sites; patients who received AV grafts as initial access were more likely to undergo additional access creation procedures when compared to those who received AV fistulas (*p* < 0.0001; Table 2).

## Discussion

This study supports the current ethos relevant to vascular hemodialysis access. Specifically, current KDOQI guidelines indicate AV fistulas are superior to AV grafts in functional patency and post-operative complications and should be considered first for initial access creation.^9^ In this study, we further investigate if patients with pre-existing diabetes mellitus alters this current clinical practice. In short, diabetic patients still had worse mid-term functional access when receiving AV grafts as their first access creation for hemodialysis, including shorter functional patency times. Diabetic patients who received AV grafts were also more likely to be older, female, non-Hispanic black, and have used central venous catheters more often for dialysis access prior to AV access creation. Post-operatively, these patients had higher rates of surgical wound infections when compared to matched diabetic patients who received AV fistulas. These results reflect other reports of vascular functional access in contemporary literature; a meta-analysis of hemodialysis vascular access in all patients noted lower rates of 2-year functional access in patients with AV grafts (40%), with even lower rates of functional access in diabetic patients.^10^ However, there have been sparsely mixed results in functional access for hemodialysis in diabetic patients; some reports indicate no association between access type and post-operative complications, including surgical wound infections,^10,11^ while another retrospective report suggests that diabetic patients fare better with initial AV graft creation versus central venous catheter placement.^12^

Within our study, there were significant differences in demographics between patients who received AV fistulas versus AV grafts for their first hemodialysis access. The most striking difference was race, as there were significantly more non-Hispanic black patients who received AV grafts versus AV fistulas. Others have detailed disparities based on race in hemodialysis as well, including reports of worse AV fistula successful implantation and functional access for non-Hispanic black patients^13^ as well as higher early failures of AV grafts in non-Hispanic black patients compared to non-Hispanic white patients.^14^ Along with these results, we also indicated higher rates of pre-operative immobility and worse living status for patients who received AV grafts. As it has been long accepted that race is a proxy for systemic healthcare disparities and other social determinants of health, these results together indicate that this patient population was more often at a higher medical disadvantage than their AV fistula counterpart, which could explain their worse outcomes overall, even with propensity score matched results.

Prior to AV creation surgery, patients needing chronic hemodialysis may be candidates for central venous catheter (CVC) placement. Proper care and management of the CVC is essential; the risk of bacteremia increases drastically with continued CVC use, especially when patients utilize them for greater than 3 months before AV access creation.^12,15–17^ However, in retrospective chart reviews, there was no discernable difference in CVC related infections in diabetic patients compared to the general population, especially in severity of infection.^16^ Nonetheless, the risk of infection and bacteremia does not disappear when patients receive AV access, either fistulas or grafts. According to a retrospective study, diabetic patients made up half of all patients who had local infections to their AV grafts.^18^ The etiology of this increased risk is theorized to be related to additional interventions after initial placement of AVGs as well as increased opportunities for colonization of abandoned AVGs.^17^ In our study, diabetic patients who received AVGs were more likely to use CVCs for hemodialysis, which predisposes for infection risk before AV creation. Albeit a small difference in our study, this result contrasts the aforementioned literature findings, suggesting that larger datasets, like the VQI, are more apt to tease out small differences in the association of access type and catheter-associated infections. Diabetic patients, in our study, who received AVGs were also approximately four times more likely to have a surgical wound infection compared to diabetic patients who received AVFs, which corroborates previously published studies.^10,18^

We also report that all diabetic patients who received AVGs had higher prescription rates of anticoagulants, but not aspirin. Lower rates of discharge anticoagulant usage, including warfarin, rivaroxaban, and dabigatran, was identified in a recent retrospective VQI study to be associated with higher rates of post-operative wound complications, especially in smokers and diabetic patients.^19^ However, this study found no reportable difference in long-term functional access in their patient population. This is surprisingly different to our results, as our diabetic patient cohort who received AVGs had higher discharge anti-coagulation (only including warfarin, rivaroxaban, and dabigatran) but still had worse long-term functional access of than those patients who received AVFs. This discrepancy again highlights the significance of studying large populations of patients with similar pathophysiology like diabetes mellitus in a single study, as it may be best to decipher these considerably unique complications in an effort to optimize best clinical practices for this patient population.

There are many factors that contribute to functional long-term functional access for initial AV access, including initial vessel size, age, and co-morbidities.^20^ In particular, a multicenter prospective cohort study found that the AVF functional access rate at 2 years was 75%, which included a majority of patients who were diagnosed with diabetes.^21^ These reports identified much higher functional access rates in general to that of AVGs, as a meta-analysis identified primary functional access at 2 years was as low as 40% for AVG.^12^ This correlated to an increase in mortality in patients who receive AVGs, even when stratified to age, race, gender, and diabetes status, among others. As diabetes is a premediating condition to chronic kidney disease and need for hemodialysis, it is difficult to independently assess the risk of AVG functional access with diabetic status alone; nonetheless, in our study, we confirm these previously published results by identifying lower AVG functional access rates in diabetic patients, which corresponds with worse post-operative outcomes. Therefore, we would advocate for the use of AVFs in diabetic patients for their initial access as much as possible, to prevent subsequent infection and early surgical intervention to maintain hemodialysis access.

There are several limitations to this study, including its retrospective nature and clarity of raw data which affects the main conclusions. As a retrospective study utilizing a national de-identified database, there will be inherent weaknesses to the conclusions we can make, due namely to our inability to control for unknown confounders. In addition, we opted to only utilize a patient’s first AV access creation procedure, as it decreased the substantial confounding variables responsible for additional surgical interventions, including inherent deterioration of patient’s health, worsening innate kidney function, and utilization of non-first choice access sites, which may lead to decreased rates of full maturation. However, in practice, this is usually not the case; many patients require continuous surgical optimization to re-establish hemodialysis access. Therefore, our results may not be as useful in clinical scenarios other than initial AV access creation. Finally, due to low patient data, we were unable to analyze endovascular AVFs in this study, which could have enhanced this work significantly given that diabetes is such a significant comorbidity for continued AV functional access. Nonetheless, future studies should be conducted on functional access for endovascular AVFs, including an investigation on how different co-morbidities including diabetes contributes to pitfalls in successful long-term usage for hemodialysis.

## Conclusions

In summary, diabetic patients who receive AV grafts for hemodialysis were more likely to be older, female, non-Hispanic black, and utilize indwelling catheters for hemodialysis before access creation compared to diabetic patients who received AV fistulas. Post-operatively, diabetics who received AV grafts had higher rates of surgical site wound infections. Ultimately, diabetic patients who received AV grafts had lower rates of mid-term functional patency, albeit with shorter time-to-use their index access for hemodialysis. Based on our study, we advocate for utilization of autogenous vein whenever possible by vascular surgeons for initial AV fistula creation in diabetic patients to prolong primary functional access and prevent post-operative complications. Alternatively, patients who require AV grafts may benefit from additional post-operative support to facilitate continued functional hemodialysis access and prevent post-operative complications like wound infections.
